# Human cardiac progenitor cell activation and regeneration mechanisms: exploring a novel myocardial ischemia/reperfusion in vitro model

**DOI:** 10.1186/s13287-019-1174-4

**Published:** 2019-03-07

**Authors:** Maria J. Sebastião, Margarida Serra, Rute Pereira, Itziar Palacios, Patrícia Gomes-Alves, Paula M. Alves

**Affiliations:** 1 Animal Cell Technology Unit, iBET, Instituto de Biologia Experimental e Tecnológica, Oeiras, Portugal; 2 ITQB-NOVA, Instituto de Tecnologia Química e Biológica António Xavier, Universidade Nova de Lisboa, Oeiras, Portugal; 3 Coretherapix, S.L.U (Tigenix Group, Takeda), Parque Tecnológico de Madrid, Madrid, Spain

**Keywords:** Cardiac progenitor cells, myocardial infarction, Myocardial ischemia reperfusion injury, Proteomics, Ischemia-reperfusion injury

## Abstract

**Background:**

Numerous studies from different labs around the world report human cardiac progenitor cells (hCPCs) as having a role in myocardial repair upon ischemia/reperfusion (I/R) injury, mainly through auto/paracrine signaling. Even though these cell populations are already being investigated in cell transplantation-based clinical trials, the mechanisms underlying their response are still poorly understood.

**Methods:**

To further investigate hCPC regenerative process, we established the first in vitro human heterotypic model of myocardial I/R injury using hCPCs and human-induced pluripotent cell-derived cardiomyocytes (hiPSC-CMs). The co-culture model was established using transwell inserts and evaluated in both ischemia and reperfusion phases regarding secretion of key cytokines, hiPSC-CM viability, and hCPC proliferation. hCPC proteome in response to I/R was further characterized using advanced liquid chromatography mass spectrometry tools.

**Results:**

This model recapitulates hallmarks of I/R, namely hiPSC-CM death upon insult, protective effect of hCPCs on hiPSC-CM viability (37.6% higher vs hiPSC-CM mono-culture), and hCPC proliferation (approximately threefold increase vs hCPCs mono-culture), emphasizing the importance of paracrine communication between these two populations. In particular, in co-culture supernatant upon injury, we report higher angiogenic functionality as well as a significant increase in the CXCL6 secretion rate, suggesting an important role of this chemokine in myocardial regeneration. hCPC whole proteome analysis allowed us to propose new pathways in the hCPC-mediated regenerative process, including cell cycle regulation, proliferation through EGF signaling, and reactive oxygen species detoxification.

**Conclusion:**

This work contributes with new insights into hCPC biology in response to I/R, and the model established constitutes an important tool to study the molecular mechanisms involved in the myocardial regenerative process.

**Electronic supplementary material:**

The online version of this article (10.1186/s13287-019-1174-4) contains supplementary material, which is available to authorized users.

## Background

Acute myocardial infarction (AMI) is still a major cause of death in the world [1]. AMI consists on the cessation of blood flow, causing oxygen and nutrient supply imbalance, leading to myocardial tissue damage, with loss of cardiomyocytes (CMs). For AMI patients, the intervention of choice is immediate myocardial reperfusion with restoration of blood flow. However, this process can aggravate the damage, as the increase of molecular oxygen levels occurs at a toxic rate (ischemia-reperfusion (I/R) injury), contributing to up to 50% of the final scar tissue size [2]. Current treatments are successful in reducing immediate mortality but do not avoid the subsequent scarring and degeneration of myocardium tissue with loss of contractile function, often leading to chronic heart failure (CHF), a highly fatal condition in which the only available clinic option is heart transplant [3].

Regenerative medicine-based strategies for infarcted myocardium include autologous and allogeneic cell therapies. Several cell types were already applied in clinical trials, including bone marrow-derived mesenchymal stromal cells (e.g., REPAIR-ACS- NCT00711542, BOOST-NCT00224536), adipose tissue-derived mesenchymal stromal cells (e.g., ADVANCE- NCT01216995, and APOLLO- NCT00442806), and cardiac progenitor cells (CPCs) (e.g., SCIPIO- NCT00474461, CADUCEUS - NCT00893360, and CAREMI- NCT02439398). For all cell types, clinical trials have demonstrated some physiological improvements but very low cell retention after some weeks, suggesting that the overall beneficial effect of transplanted cells is due to paracrine modulation rather than differentiation and functional integration in the tissue [4]. In fact, novel strategies that focused on the induction of the endogenous heart regenerative potential, such as direct growth factor administration, have shown to induce tissue regeneration by reduction of fibrosis, induction of angiogenesis, inhibition of apoptotic processes, and recruitment of endogenous CPCs [5, 6].

Although in low percentages, endogenous CPCs seem to play an important regenerative role in cardiac homeostasis and in response to physiological stress and I/R injury. While the physiological alterations undergone by CMs during I/R have been extensively covered, the mechanisms by which CPCs exert their protective role are still not well defined. Upon injury, there are increased levels of signaling growth factors and cytokines released in the myocardium [7, 8]. Such signals have already been proposed to trigger CPC proliferation, differentiation, migration to the site of injury, and growth factor secretion that together have an effect on cardiomyocyte protection, reduction of inflammation, and reduction of scar tissue size, which has already been documented in several animal studies [9, 10]. Nevertheless, some doubts and controversy still exist regarding the identity and ability of CPCs to generate new CMs upon injury [11, 12]. Such concerns have recently been enhanced with the retraction of several CPC studies from Piero Anversa’s lab [13]. The hCPCs employed in this work are not the same population involved in this controversy and were isolated and cultured using a different protocol (Patent WO2014141220A1), which was also used for the isolation of the hCPC population recently evaluated for the allogeneic treatment of AMI in CAREMI clinical trial (NCT02439398). Importantly, these cells were already extensively characterized at the immunomodulatory [14–17], total proteome [18], membrane proteome [19, 20], and secretome [21] levels. The regenerative benefit of these cells was studied in vivo in pig AMI animal model [22, 23].

Mainly due to the lack of relevant human models, the role of CPCs in I/R injury has been studied in vitro with murine cells. There are relevant gaps between murine and human cardiac physiology both in vitro and in vivo, such as different functionality of CM ion channels and a higher tolerability to drugs in mice and mice cells. Such differences can cause misinterpretation of the results and have already been pointed as one of the causes of high drug attrition rates [24].

Aiming at filling the gap between murine and human in vitro models, in this work we have established the first in vitro human cell-based myocardial I/R injury model, using human CPCs (hCPCs) and human-induced pluripotent stem cell-derived CMs (hiPSC-CMs). The goal of our work is to use this co-culture model as a tool to better understand and characterize hCPC response to I/R injury, and its effect on CM death and survival upon injury. Our model was able to recapitulate important features of AMI, namely CM death, a paracrine protective effect of CPCs in CM survival, and CPC proliferation activation. For the first time, CXCL6, a cytokine with documented angiogenic properties, already identified as having an important regenerative role in both mesenteric and myocardial infarction was found to be highly secreted by hCPCs upon I/R injury in the co-culture condition. Moreover, we also demonstrated higher angiogenic potential of the co-culture supernatant upon ischemia. Human CPC whole proteome analysis showed that upon injury, and in the presence of hiPSC-CMs, there was an enrichment in proteins involved in pathways and functions related with cell proliferation, paracrine signaling, stress response, and regeneration processes when comparing to control and mono-culture conditions.

## Methods

### Cell culture

CPCs were obtained from human right atria appendage myocardial tissue, isolated, and characterized as described elsewhere [14]. Cells were cultured at 37 °C in humidified incubators (5% CO_2_, 3% O_2_) in expansion medium (ExpM) composed by DMEM:F12: Neurobasal medium (1:1), supplemented with 1% penicillin streptomycin, 10% fetal bovine serum embryonic stem cell-qualified, N2 supplement (1×), B27 supplement (1× ), 0.9 mM l-glutamine, 50 μM β-mercaptoethanol (Sigma), insulin transferrin selenium (0.5× ), 10 ng/mL bFGF, 20 ng/mL EGF-I, and 30 ng/mL IGF-II (Prepotech) (all percentages in *v*/*v*). Medium was replaced by 50% every 3 days. Cells were subcultured when about 80% confluent using Tryple™ Select Enzyme for 5 min at 37 °C. All cell culture reagents were purchased from Gibco, Life Technologies unless otherwise stated.

Human iPSCs (DF19-9-11 T.H, WiCell) were cultured and differentiated to CMs (hiPSC-CMs) as previously described [25, 26]. Using this protocol, monolayer cultures composed of > 90% of hiPSC-CMs were obtained after 15 days. To further improve the maturation state of this cell population, hiPSC-CMs were cultured for additional 10 days in Pluricyte Medium (NCardia), as described elsewhere [27]. Cells were maintained at 37 °C in humidified incubators (5% CO_2_, 95% air).

### Ischemia/reperfusion injury setup

I/R experiments were performed with the following: mono-cultures of hCPCs (plated at 2 × 10^4^ hCPCs/cm^2^), mono-cultures of hiPSC-CMs (plated at 1–1.5 × 10^5^ hiPSC-CMs/cm^2^), and co-cultures of the two cell types (hCPCs:hiPSC-CMs 1:10–1:20) using Transwell® permeable insert supports (0.4 μm pore size, Corning). The insert supports are semi-permeable polyester membranes separating both cell types allowing paracrine interaction. hCPCs were seeded in transwell supports (1.2 × 10^4^ hCPCs/insert and positioned above hiPSC-CM culture dishes (1.2–2.4 × 10^5^ hiPSC-CMs/ well) at the beginning of the ischemia phase, allowing a paracrine interaction between the two cell types during all the sequence of I/R injury.

**Fig. 1:**
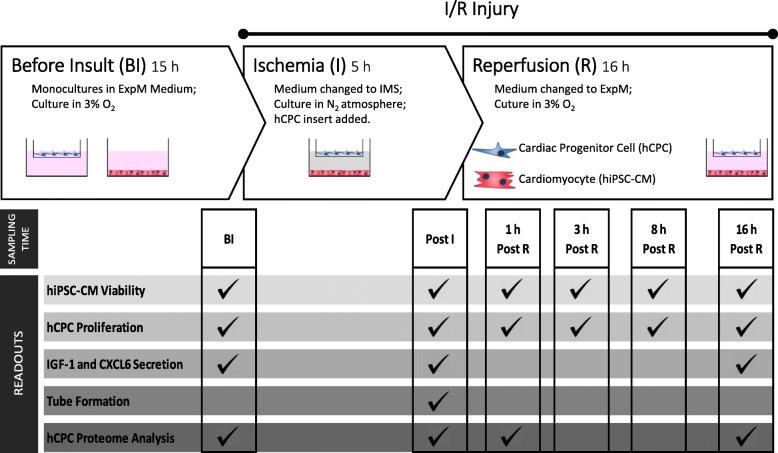
Schematic representation of ischemia/reperfusion injury experimental setup. Ischemia was mimicked by replacing expansion medium by ischemic mimetic solution (IMS) and by culturing cells in a N_2_ gaseous environment. After 5 h of ischemia, reperfusion was mimicked by re-establishing normoxic culture conditions (ExpM at 3% O_2_). I/R setup was performed using mono-cultures of hiPSC-CMs, mono-cultures of hCPCs, and co-cultures of the two cell types using transwell permeable inserts. The impact of the I/R was evaluated regarding hCPC proliferation, hiPSC-CM viability, hCPC whole proteome, and secretion of IGF-1 and CXCL6 factors in different time points: (BI—before injury; Post I—post ischemia; Post R—post reperfusion). Control cultures were done in parallel (using normoxic conditions)

All cell populations were cultured in ExpM at 3% O_2_ (myocardial physiologic normoxia) in humidified incubators (5% CO_2_, 95% air) at least 15 h before I/R experiments. I/R injury experimental setup is illustrated in Fig. 1. Briefly, ischemia was mimicked by replacing ExpM by ischemic mimetic solution (IMS; in mM: NaCl, 135; KCl, 8; MgCl_2_, 0.5; NaH_2_PO_4_, 0.33; HEPES, 5.0; CaCl_2_, 1.8; Na^+^-lactate, 20; pH 6.8) [28] and by placing cells in a N_2_ gaseous environment at 37 °C. After 5 h of ischemia, reperfusion was mimicked by re-establishing control culture conditions (ExpM at 3% O_2_). Control cultures for all culture setups were maintained in parallel (ExpM at 3% O_2_). The impact of I/R injury was evaluated regarding hCPC proliferation, hiPSC-CM viability, secretion of growth factors, and hCPC whole proteome analysis.

### Total cell number

hCPC number was accessed by nuclei count with crystal violet solution staining. Briefly, cells were resuspended in lysis buffer (0.1% Triton X-100 in 0.1 M citric acid) directly in culture wells/transwells and incubated at 37 °C for at least 48 h. Nuclei were stained with crystal violet dye (0.1% *v*/*v* in lysis buffer) and the total number of nuclei counted in a Fuchs-Rosenthal hemocytometer chamber. Fold increase in hCPC number was calculated as the ratio between the cell number at the experimental time point assayed and cell number before I/R injury.

### Cell viability

hiPSC-CM viability was assessed by cell membrane integrity analysis: cell monolayers were incubated with 20 μg/mL fluorescein diacetate (FDA), that stains viable cells, and 10 μg/mL propidium iodide (PI), a membrane impermeable DNA-dye that stains non-viable cells, in DPBS for 2–5 min. Samples were then observed under a fluorescence microscope (DMI 6000, Leica Microsystems GmbH).

hiPSC-CM viability was further assessed using the metabolic indicator PrestoBlue® Cell Viability Reagent (Life Technologies), according to the manufacturer’s recommendation. Briefly, cells were incubated with ExpM containing 10% (*v*/*v*) PrestoBlue®, for 1 h. Supernatant’s fluorescence was measured in 96-well plates using a microwell plate fluorescence reader (Infinite 200 PRO NanoQuant TECAN). Values obtained were normalized by the initial values obtained before I/R injury.

### Immunofluorescence microscopy

Cell monolayers were washed with DPBS and fixed in 4% (*w*/*v*) paraformaldehyde (PFA) and 4% (*w*/*v*) sucrose in DPBS for 20 min. Afterwards, cells were permeabilized for 10 min in 0.1% (*v*/*v*) Triton X-100 in DPBS and blocked with 0.2% (*v*/*v*) fish skin gelatin (FSG) in DPBS for 30 min, at room temperature (RT, 18–20 °C). Cells were then incubated with primary antibodies diluted in 0.125% (*v/v*) FSG, 0.1% (*v/v*) Triton X-100 for 2 h at RT. Cells were washed with DPBS and incubated with secondary antibodies diluted in 0.125% (*v/v*) FSG, 0.1% (*v/v*) Triton X-100 for 1 h at RT in the dark. The following primary antibodies were used: α-sarcomeric actinin (1:200, Sigma), CD26 (1:80, Thermo Fisher), and Ki-67 (1:200, Abcam).

### Flow cytometry

After a 5 min dissociation step with TrypLE™Select at 37 °C, cells were washed with DPBS by centrifugation and a total of 2–3 × 10^5^ cells were used per analysis. For membrane markers, cells were incubated with the primary antibody for 1 h and with the secondary antibody for 30 min at 4 °C in DPBS with 5% (*v*/*v*) FBS. For intracellular markers, cells were permeabilized using the Inside Stain Kit (Miltenyi Biotec) according to the manufacturer‘s instructions. The following primary antibodies were used: SirPα/β (1:20, CD172a/b-PE, BioLegend), Troponin T (1:200, TnT, Thermo Scientific), CD105 (1:20,BD Pharmingen), CD166 (1:20,BD Pharmingen), CD44 (1:5, eBiosciences), CD11b (1:10, AbDSerotec), CD34 (1:5, BD Pharmingen), CD45 (1:5, BD Pharmingen), and isotype controls mouse igG1k (1:5, BD Pharmingen), mouse IgG1 (1:2.5,Santa Cruz Biotechnologies), and rat IgG2b (1:5, eBiosciences). Cells were analyzed in a CyFlow® space (Partec GmbH) instrument, registering at least 10,000 events/sample.

### Quantification of growth factors

Quantification of growth factors CXCL6 and IGF-1 in cell conditioned medium was performed by ELISA Human Quantikine ELISA kit (R&D Systems), according to the manufacturer’s instructions. Optical density was measured in 96-well plates using a microwell plate reader (Infinite 200 PRO NanoQuant TECAN). The specific rate of growth factor secretion was estimated according to the following equation: ${q}_{\mathrm{GF}}=\frac{\Delta {C}_{\mathrm{GF}}}{C_{\mathrm{cell}}\times \Delta t}$

where Δ*C*_GF_ (g/L) is the variation in growth factor concentration during the time period Δ*t* (*h*) and *C*_cell_ is the concentration of cells (cell/L).

### HUVEC culture and tube formation assay

Human umbilical vein endothelial cells (HUVECs, Lonza ref. 2517A) were cultured at 37 °C in humidified incubators (5% CO_2_, 3% O_2_), in 0.1% gelatin-coated plates with Endothelial Cell Growth medium 2 (ECGM2, PromoCell). Medium was replaced every 3 days. Cells were subcultured when about 90% confluent using 0.5% Trypsin-EDTA for 7 min at 37 °C.

Tube formation assay was performed according to Pedroso et al. [29]. Briefly, ice-cold undiluted Matrigel (Growth factor Reduced, BD Biosciences) (1.97 mg/cm^2^) was used to coat 96-well plates and incubated for 40 min at 37 °C to allow the Matrigel to solidify. HUVECs were seeded at a density of 5.5 × 10^4^ cells/cm^2^ and incubated with the conditioned media from I/R experiments. ECGM2 was used as positive control for tube formation. At least four independent images were acquired per condition after 4 h of incubation and the morphological aspects of the tube network were quantified using the ImageJ angiogenesis analyzer plugin, including total branching length (sum of length of the trees composed from segments and branches), total segment length (sum of length of the segments), and number of nodes [30].

All cell culture reagents were purchased from Gibco, Life Technologies unless otherwise stated.

### Whole proteome analysis

hCPCs were harvested and washed twice with DPBS by centrifugation. Supernatants were discarded and cell pellets were placed at − 80 °C until further analysis. Proteins were extracted, quantified, and processed from cell pellets as described elsewhere [31]. Two biological replicates with three technical replicates per time point were run. Protein samples were analyzed by NanoLC–MS/MS using TripleTOF 6600 (ABSciex). External calibration was performed using beta-galactosidase digest (ABSciex). The 40 most intense precursor ions from the MS spectra were selected for MS/MS analysis. Data were acquired with the Analyst software TF 1.7 (ABSciex). The raw MS and MS/MS data were analyzed using Protein Pilot Software v.5.0 (ABSciex) for protein identification. The search was performed against the Swissprot protein database with taxonomic restriction to *Homo sapiens*. Protein identification was considered when unused scores were greater than 1.3 (95% confidence). Analysis of the protein lists was performed using Venny 2.1 (http://bioinfogp.cnb.csic.es/tools/venny/) and ingenuity pathway analysis (IPA, Qiagen). Statistically significant representation of biological functions and canonical pathways was identified based on IPA *p* value. This probability score is calculated taking into account the total number of proteins known to be associated with a given function or pathway, and their representation in the experimental dataset. IPA’s calculated *p* value is displayed as –log (*p* value). All proteomic data have been deposited in the ProteomeXchange Consortium (http://proteomecentral.proteomexchange.org) via the PRIDE partner repository with the dataset identifier PXD008156.

### Statistical analysis

Statistical analyses were performed with GraphPad Prism6 (GraphPad Software Inc.). All data are shown as mean with standard deviation. Differences in hCPC fold increases (*n* = 3/5), differences in hiPSC-CM viability percentages (*n* = 2/3), differences in specific growth rate secretion of CXCL6 and IGF-1 (*n* = 3), and differences in tube formation (*n* = 3/4) were analyzed by parametric one-way ANOVA Tukey test. Individual *p* values for each comparison were obtained using the multiplicity adjusted *p* value test. Differences for ki67 expression percentage were analyzed by parametric Student’s *t* test with Welch’s correction. *p* values below 0.05 were considered significant.

## Results

In this study, we developed an in vitro I/R injury co-culture model with hCPCs and hiPSC-CMs, using transwell inserts to allow paracrine communication between the two cell types.

Identity of hCPCs was demonstrated by the expression of hCPC cell-surface-specific markers by flow cytometry. As demonstrated before [18], this cell population is negative for CD34, CD45, CD11b, and CM marker cTnT and displays a high percentage of cells positive for adult stem cell markers CD44 (97.2% ± 2.2), CD105 (95.5% ± 1.7), and CD166 (75.1% ± 13.3) (Additional file 1: Figure S1A). The expression of CD26 (DPP4), recently found to be selectively upregulated in hCPCs when compared to human mesenchymal stem cells and human dermal fibroblasts (Tóran J, López J, Gomes-Alves P, et al., submitted), was also detected by immunostaining (Additional file 1: Figure S1B).

During the establishment of the I/R injury setup, two different types of hiPSC-CMs were tested: (i) hiPSC-CMs differentiated as previously described [25, 26] and (ii) the same cells with an extra maturation step [27] (please see material and methods section for details). With the first hiPSC-CMs tested (without the extra maturation step), cell viability was not affected by the injury setup neither in mono-culture nor in co-culture with hCPC conditions (Additional file 2: Figure S2). These results suggest that these cells were not metabolically mature enough, not reflecting the phenotype of adult CMs found in the heart. Such results are in accordance with the findings that point to a relevant role of CM maturation stage on cell survival upon I/R injury. In fact, primary embryonic human CMs are resistant to hypoxia, while primary adult human CMs are highly dependent on an adequate oxygen supply, which might be related with the different metabolic phenotypes between the different developmental stages [32, 33]. hiPSC-CMs with an extra maturation step were therefore used in the following studies, since they better reflected the typical loss of cell viability during AMI.

hiPSC-CM typical cardiac markers were not altered in our assay conditions (ExpM at 3% O_2_, representing myocardial physiological normoxia (Khan et al. [34])) when compared to the cell’s maturation culture conditions (Pluricyte® CM medium, 21% O_2_), presenting a high percentage of cells positive for the cardiac-specific proteins SIRPA (72.9% ± 5.2) and cTnT (92.6% ± 2.9) (Additional file 3: Figure S3A) and the characteristic striated pattern of α-sarcomeric actinin (Additional file 3: Figure S3B).

The heterotypic model was subjected to I/R injury (Fig. 1) and analyzed in terms of hCPC proliferation, hiPSC-CM viability, secretion of key growth factors, angiogenic potential, and hCPC whole proteome. Co-cultures were compared with mono-cultures of the two cell types and controls without injury.

### hCPC proliferation is activated upon I/R in presence of hiPSC-CM

hCPC proliferation was evaluated in control (CTL) and injury conditions in mono- and co-culture with hiPSC-CMs.

**Fig. 2:**
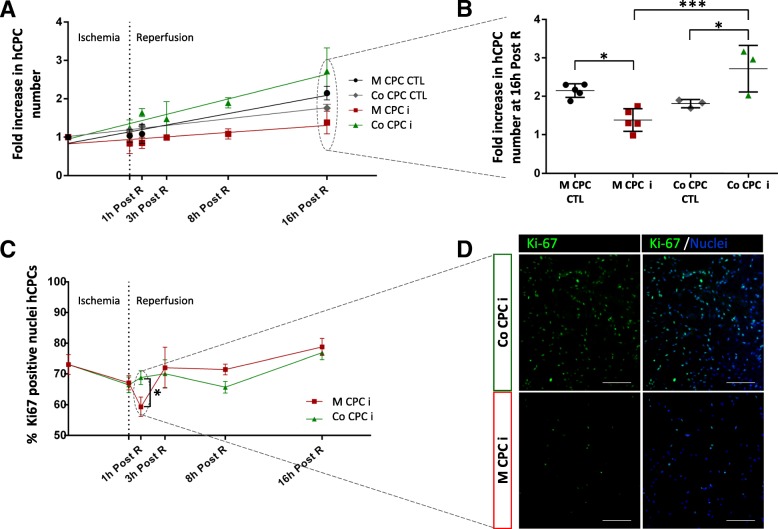
Impact of I/R injury conditions on hCPC proliferation**. a** Fold increase in hCPC cell number. **b** Fold increase in hCPC cell number at 16 h post reperfusion. **c** Quantification of Ki67^+^ hCPCs by immunostaining. **d** Representative culture imaging of Co CPC i and M CPC i labeled with Ki67 at 1 h Post R. Black circles: mono-culture CTL (M CPC CTL); grey diamonds: co-culture CTL (Co CPC CTL); red squares: mono-culture hCPC insult (M CPC i); green triangles: co-culture hCPC insult (Co CPC i). Post R: post reperfusion. *p < 0.05; **p < 0.01; *** *p* < 0.005. Fold increase was calculated as the ratio between the cell number at the experimental time point assayed and cell number before injury

hCPCs in co-culture with hiPSC-CMs showed a significant higher fold increase at 16 h post reperfusion when compared to the respective co-culture control (2.7 ± 0.6 vs 1.8 ± 0.1, respectively, *p* = 0.0396) and when compared with hCPCs in mono-culture in injury condition (2.7 ± 0.6 vs 1.4 ± 0.3, respectively, *p* = 0.0009) (Fig. 2a, b).

To further confirm the differences observed in the proliferation profile between hCPCs subjected to injury in both mono- and co-culture conditions, hCPCs were labeled with Ki-67, a marker specific for active proliferating cells. Although for all conditions tested the percentage of Ki-67^+^ hCPCs was between 60 and 80%, there was a significant decrease of Ki-67^+^ hCPCs at 1 h post reperfusion in mono-culture in comparison with co-culture conditions (59.3% ± 3.14 vs 68.8% ± 2.3, respectively, *p* = 0.0163) (Fig. 2c, d), which might explain the lower cell proliferation observed in this condition. These findings suggest that injury diminishes hCPC proliferation capacity, especially in the early reperfusion period. Such effect is, however, prevented when the co-culture model was used.

### hCPC exert a paracrine protective effect on hiPSC-CM upon I/R

**Fig. 3:**
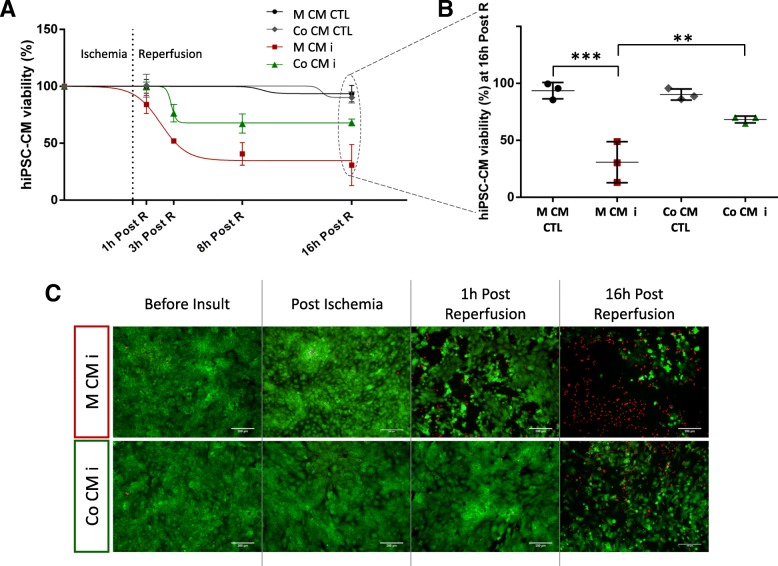
Effect of I/R injury on hiPSC-CM viability. hiPSC-CM viability was assessed by PrestoBlue® assay (**a**, **b**) and by cell staining with FDA (live cells, green) and PI (dead cells, red), scale bars 200 μm (**c**). Black circles: mono-culture hiPSC-CM CTL (M CM CTL); grey diamonds: co-culture hiPSC-CM CTL (Co CM CTL); red squares: mono-culture hiPSC-CM insult (M CM i); green triangles: co-culture hiPSC-CM insult (Co CM i). Post R: post reperfusion. * *p* < 0.05; ** *p* < 0.01; *** *p* < 0.005

The impact of I/R injury conditions in hiPSC-CM viability was evaluated for mono- and co-culture conditions. A decrease in viability was observed for hiPSC-CM upon injury when compared to CTL, triggered after the first hour of reperfusion (Fig. 3a). hCPCs exerted a paracrine protective effect on hiPSC-CMs in the co-culture condition, with a higher viability ratio across all reperfusion time points assayed when compared with the mono-culture condition, as confirmed by PrestoBlue® Cell Viability Reagent results (Fig. 3a, b) and by cell staining with live/dead cell dyes (Fig. 3c). At 16 h post reperfusion, hiPSC-CM subjected to injury in co-culture conditions presented significantly higher viability when compared to hiPSC-CM in mono-culture (68.4% ± 3.0 vs 30.8% ± 17.9,respectively, *p* = 0.008) (Fig. 3b). When comparing to respective controls, at 16 h post reperfusion, hiPSC-CMs in mono-culture showed a significant decrease of 62.8% in viability, while in co-culture, hiPSC-CM viability dropped only by 21.9%.

### Secretion of key growth factors is upregulated in I/R

To further access the paracrine effect observed in the co-culture condition, the secretion of two key proteins with documented cardiac regenerative properties was quantified in our I/R system: chemokine ligand 6 (CXCL6 or GPC-2) and insulin-like growth factor 1 (IGF-1) [6, 35–37].

**Fig. 4:**
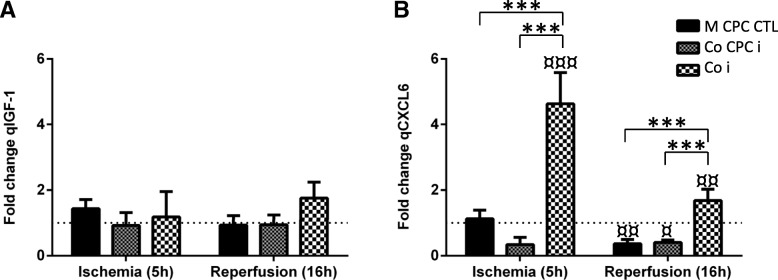
Specific rates of CXCL6 and IGF-1 secretion during ischemia and reperfusion**. a** qIGF-1 measured in conditioned media of mono-culture hCPC CTL (M CPC CTL); mono-culture hCPC insult (M CPC i) and co-culture hCPC:hiPSC-CM insult (Co i). **b** qCXCL6 measured in conditioned media of mono-culture hCPC CTL (M CPC CTL); mono-culture hCPC insult (M CPC i) and co-culture hCPC:hiPSC-CM insult (Co i). Specific growth factor secretion rates were normalized to the values before insult (dashed line). * *p* < 0.05; ** *p* < 0.01; *** *p* < 0.005; ^¤^ *p* < 0.05; ^¤¤^ *p* < 0.01; ^¤¤¤^ *p* < 0.005; ^¤¤¤¤^ *p* < 0.0001 vs before insult

Regarding IGF-1, our data shows that both hCPCs and hiPSC-CMs secrete IGF-1 either in mono- and co-culture conditions (data not shown for hiPSC-CM mono-cultures). IGF-1 is secreted and recognized by both cell types [5, 37, 38]; therefore, it was not possible to isolate and quantify how much IGF-1 each cell population secreted in the co-culture condition, neither if there was an uptake of IGF-1 by hiPSC-CM or hCPC. We could not detect significant changes in IGF-1 secretion upon injury neither in hCPC mono-cultures nor in co-cultures with hiPSC-CMs (Fig. 4a).

CXCL6 seems to be selectively secreted by hCPC, as it was not identified in either control or injury conditioned medium of mono-cultures of hiPSC-CMs (data not shown). However, in the co-culture condition, there is a significant increase in CXCL6 cell-specific secretion rate in both phases of injury, more pronounced during ischemia (Fig. 4b). In hCPC mono-culture conditions, we observed some alterations in the CXCL6 cell-specific secretion rates during injury, but such rates do not differ between control and injury (Fig. 4b).

**Fig. 5:**
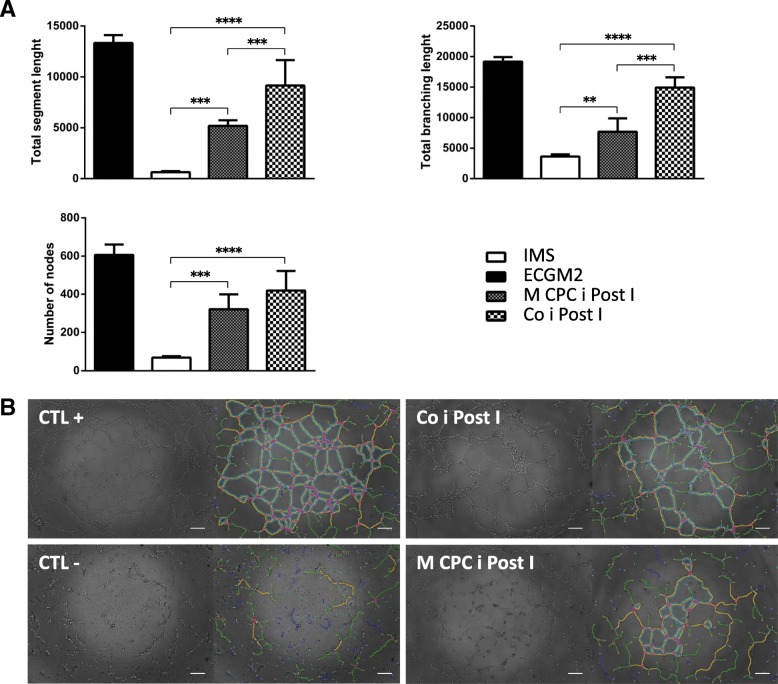
Angiogenic functional evaluation of I/R conditioned medium. Ischemic mimetic solution (IMS), endothelial cell growth medium 2 (ECGM2), and conditioned media of hCPC mono-culture post ischemia (M CPC i Post I) and of co-culture post ischemia (Co i Post I) conditions were tested for angiogenic potential by HUVECS tube formation assay. **a** Total segment length, total branching length, and number of nodes are represented. ^¤¤^ *p* < 0.01; ^¤¤¤^ *p* < 0.005; ^¤¤¤¤^ *p* < 0.0001 vs IMS. **b** Representative original images (left) and quantification by angiogenesis analyzer are shown. In the right image, there is an indication of master junctions (pink dots), master segments (yellow), meshes (light blue), branches (green), and isolated segments (blue). Scale bars 200 μm

CXCL6 is described as being an angiogenic chemokine secreted by CPCs [21]. In order to access if the I/R conditioned medium has a pro-angiogenic paracrine profile, the in vitro tube formation assay was performed. HUVECs were incubated with conditioned media from co-cultures and mono-cultures of hCPCs upon ischemic injury. As shown in Fig. 5, the angiogenic potential of the conditioned medium from the co-culture condition post ischemia is higher when comparing to the mono-culture medium, represented by a significant increase in total segment length and total branching length as well as by a higher number of nodes (Fig. 5a).

### Proteomic analysis of hCPCs reveals enrichment in key hallmark pathways and functions upon injury

**Fig. 6:**
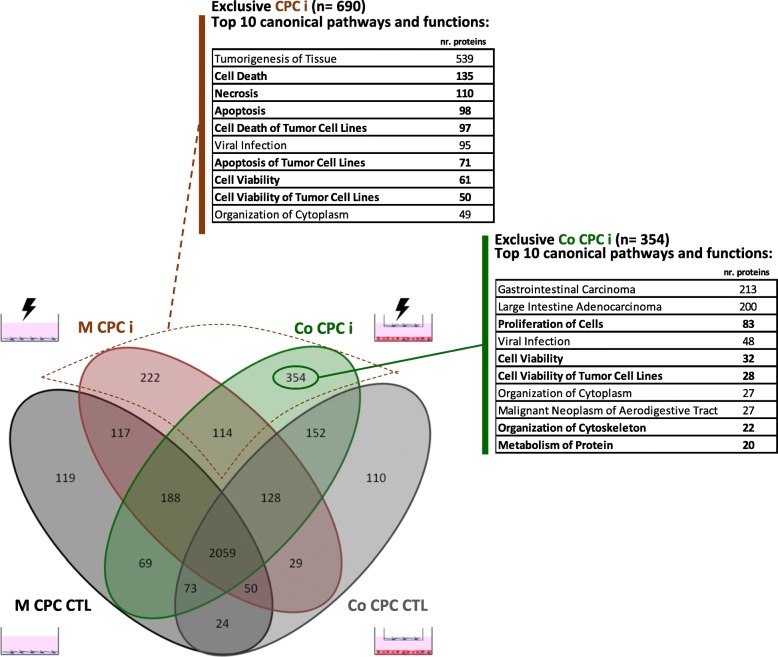
hCPC whole proteome analysis. Venn diagram illustrates the overlap between proteins identified in hCPCs in mono-culture control (M CPC CTL), co-culture control (Co CPC CTL), mono-culture insult (M CPC i), and co-culture insult (Co CPC i) conditions. Top 10 canonical pathways and functions with −log(*p* value) ≥ 1.3 and the highest number of identified proteins are highlighted for the subsets of proteins exclusively identified in hCPCs subjected to I/R injury in both mono- and co-culture conditions (CPC i, red) and for the subset of proteins exclusively identified in Co CPC i proteome (green)

To further understand the regenerative response of hCPCs to the I/R injury, whole proteome analysis of hCPCs was performed in all culture conditions. More than 3800 proteins were identified in all samples (Fig. 6). Biological canonical pathway and functions (terms) enrichment analysis was performed using IPA software (full list of scores of canonical pathways and functions analysis in Additional file 4). Our analysis was focused on terms with relevant described roles in AMI, associated with the following categories: cell proliferation, cytoskeleton organization, maintenance of cell viability, cell death, oxidative stress, paracrine signaling, regeneration, stress response, and metabolism.

Proteins exclusively identified in hCPCs subjected to I/R injury in mono- (M CPC i) and co-culture (Co CPC i) (*n* = 690) were analyzed. Within this subset, from the 10 top terms with the highest number of proteins, 7 were associated with cell death and viability (Fig. 6), suggesting that the I/R injury setup elicited an hCPC physiological response involving activation of mechanisms of cell survival.

Approximately 9% of the identified proteins (*n* = 354) were exclusively identified in hCPCs subjected to injury in co-culture conditions (Co CPC i). Within this subset, there was a high enrichment in proteins associated with cell proliferation (*n* = 83), cell viability (*n* = 32), organization of cytoplasm (*n* = 27), organization of cytoskeleton (*n* = 22), and metabolism of protein (*n* = 20), all included in the 10 top terms (Fig. 6). By exploring in more detail this subgroup of proteins (Additional file 5: Figure S4), we can highlight the identification of APOD and APOH, proteins involved in CM viability and opsonization [39], proteins associated with CXCR4 signaling (RHOB, PIK3R1, GNAQ, PRKCA, RPS6KB2), proteins associated with IGF-1 signaling (JAK1, PIK3R1, RPS6KB2, SFN, RASA1, IGFBP2), and SERPINB3, a protease inhibitor activated by hypoxia [40] with described roles in protection from oxidative damage [41], cell proliferation [42], IL-6 signaling, and unfolded protein response [43].

Moreover, several other pathways and functions relevant in I/R response were enriched in Co CPC i proteome when compared to control (Co CPC CTL) (Additional file 6: Table S1) and M CPC i (Additional file 7: Table S2), including cell proliferation via EGF-mediated pathways, pathways associated with actin filaments, with reactive oxygen species (ROS) metabolism, corticotropin-releasing hormone signaling (described has playing a major role in cell adaptation under stressful conditions [44]), and glycolysis. Paracrine signaling terms more enriched in Co CPC i proteome included IGF-1, HGF, VEGF, Oncostatin M, Neuregulin, Netrin, PDGF, IL-1, IL-2, IL-3, and IL-6 signaling pathways (Additional file 6: Table S1 and Additional file 7: Table S2), all with documented roles in myocardial I/R [45–48].

Following this first comparative analysis, a more exhaustive proteomics characterization of hCPCs in the co-culture condition was carried out. Term enrichment was compared between different time points, namely in the control situations (Co CPC CTL), immediately post ischemia period (Co CPC Post I), 1 h post reperfusion (Co CPC 1 h Post R), and 16 h post reperfusion (Co CPC 16 h Post R) (Additional file 8)

By analyzing the different time points, terms related with cell proliferation via EGF (EGF, cholecystokinin/gastrin-mediated and ERK5 signaling) were found as having an increasing representation along injury (Additional file 8, Category 1). FLT3 signaling was also identified as more enriched at 16 h post reperfusion, while cytokinesis (Additional file 8, Category 1) and IGF-1 signaling, also involved in cell proliferation activation [49] (Additional file 8, Category 5), were found as more enriched post ischemia. Regarding terms associated with cytoskeleton organization (Additional file 8, Category 2), cell invasion, extension, and CCR3 signaling (involved in eosinophils recruitment to inflammation sites [50]) were enriched after the ischemic period, while terms associated with cell movement were enriched at 1 h post reperfusion, suggesting an activation of hCPC migration and homing to injury in these early injury time points. Importantly, although several terms related with cell death and survival were found as less enriched in hCPCs upon ischemia, the same returned to their control levels during the reperfusion period (Additional file 8, Category 3).

It is worth to mention that the cell cycle regulation pathways (Additional file 8, Category 1) and DNA repair mechanisms, including the BER pathway (Additional file 8, Category 3) representation, decreased on early injury time points and recovered to values closer to control at 16 h post reperfusion. Regarding oxidative stress, terms related with degradation and metabolism of H_2_O_2_ and HIF-1a signaling were found as more enriched in hCPCs post ischemia (Additional file 8, Category 4).

IGF-1 and GM-CSF signaling was also more represented upon ischemia (Additional file 8, Category 5), while other pathways associated with cytokine/growth factor paracrine signaling and cardiac regeneration were found as more enriched in hCPCs after 16 h of reperfusion, including VEGF, Neuregulin, Oncostatin M, Jak/Stat, PDGF, IL-2, IL-3, IL-6, IL-15, and IL-22 signaling (Additional file 8, Category 5).

Stress response-associated functions such as hypersensitive reaction, endoplasmic reticulum stress (ERS), unfolded protein response (UPR), and cardiac β-adrenergic signaling were more enriched upon ischemia (Additional file 8, Category 6), while acute phase response signaling and stress response of cell representation was higher at 1 h post reperfusion. Importantly, cardiac regeneration associated functions such as cell differentiation, vasculogenesis, and angiogenesis were also found as more enriched after 16 h of reperfusion (Additional file 8, Category 6). Cysteine biosynthesis and glutathione-mediated detoxification terms, both important for protection from oxidative stress [51], were found as more enriched post ischemia (Additional file 8, Category 7). Glycolysis and oxygen consumption-related pathways was also found to be more represented upon ischemia (Additional file 8, Category 7), which might point to a compensatory mechanism to lack of oxygen availability during this phase of injury.

## Discussion

**Fig. 7:**
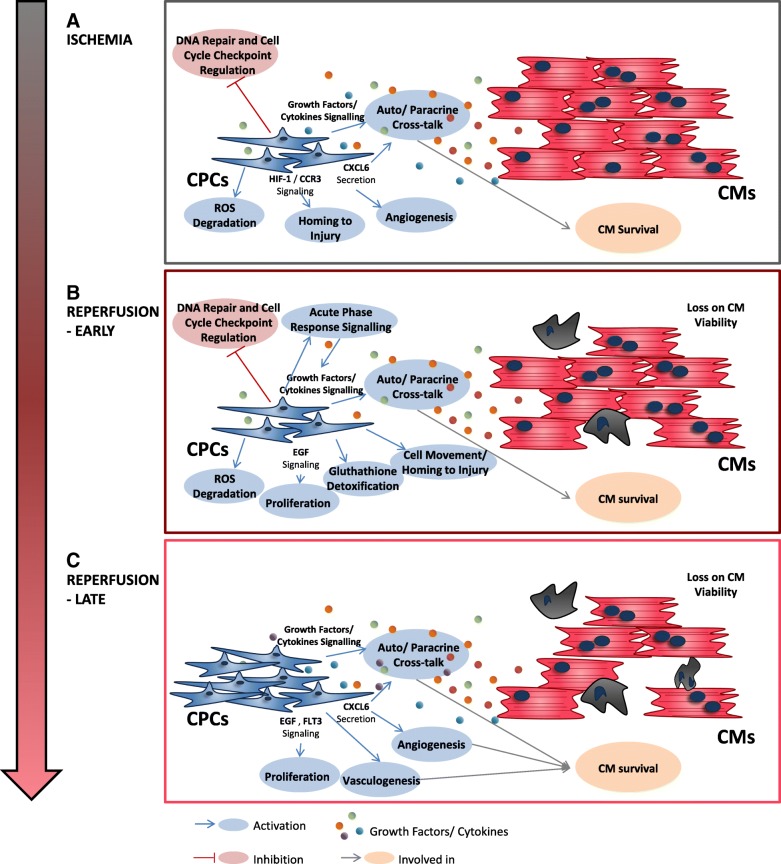
Schematic representation of proposed mechanisms involved in hCPC response to the I/R injury model**,** during **a** ischemia, **b** early (1 h post reperfusion), and **c** late (16 h post reperfusion) reperfusion phases of I/R injury

In this study, we developed an in vitro human cellular model of myocardial I/R injury with hCPCs and hiPSC-CMs that enabled us to further decipher the action mechanisms of hCPCs upon injury. The co-culture model developed recapitulates important hallmarks of I/R injury, namely CM death, CPC proliferation activation upon insult, and the protective role of CPCs on CMs. New players on hCPC regeneration response upon I/R including activation of pathways related with cell proliferation, cytoskeleton organization, maintenance of cell integrity, stress response, paracrine signaling, cardiac regeneration, and metabolism were identified based on proteome hCPC data (Fig. 7). We also showed, for the first time, an increase in the CXCL6 secretion rate by hCPCs in an I/R setting.

During the ischemic phase of injury, cells were cultured in conditions mimicking the myocardial pathophysiological state of ischemia including lack of oxygen availability, nutrient deficiency, acidosis, lactate accumulation, and hyperosmosis [28]. hCPC proteomic analysis results point to a downregulation of cell cycle regulation pathways, DNA repair mechanisms, and cell repair mechanisms during the ischemic phase of injury (Fig. 7a). In fact, downregulation of cell cycle checkpoint control and DNA repair processes has been reported as a process involved in activation of several types of quiescent adult stem cells [52]. Proteome analysis also indicates an activation of endoplasmic reticulum stress pathways, such as ERS and UPR, which have been previously associated with cellular adaptation to glucose deprivation and hypoxic stimuli [53]. Proteins associated with glycolysis and consumption of oxygen were also enriched in this time point, which might point to an adaptive response of hCPCs to the lack of O_2_, via an increase in the expression of protein machinery associated with energy production.

The ability of CPCs to differentiate into de novo CMs upon AMI is still under active discussion, with different authors defending contrary results [11, 12, 54]. In our model, hCPCs did not label for Nkx2.5, an early marker of cardiac differentiation (results not shown) neither in CTL nor in injury conditions, although an enrichment in proteins related with cell differentiation was found in hCPCs upon the ischemic phase of injury.

Although such controversy still exists regarding cardiac differentiation potential of CPCs upon injury, there is a general consensus in the field that CPCs exert a protective effect on hCMs under stress mainly due to paracrine mechanisms [37, 55], supported by extensive preclinical and clinical data indicating that transplanted CPCs do not survive neither engraft in the myocardium while physiological improvement is still registered [4]. In order to further understand the paracrine crosstalk between hCPCs and hiPSC-CMs in our model, we quantified the secretion of two key proteins: CXCL6 and IGF-1, both with documented cardiac regenerative properties. CXCL6 is an angiogenic chemokine shown to improve heart function in mice [21, 35] and identified as upregulated during mesenteric (intestinal) I/R injury [56]. This chemokine was also recently identified in the secretome of hCPCs, where addition of an anti-CXCL6 antibody inhibited the migration and angiogenic properties of CPC conditioned medium, proving the importance of this chemokine in key paracrine regenerative potential features of these cells [21]. We show for the first time an increase in the hCPC CXCL6 secretion-specific rate in a myocardial I/R injury setting, which was more pronounced during ischemia, but still significant after 16 h of reperfusion (Fig. 7a, c). These results support previous findings that show that this chemokine is upregulated in ischemic conditions via HIF-1α signaling [57]. Cardiac stem cells have been shown to home and migrate to the site of injury, a process activated by HIF-1α transcription factor and SDF-1 chemokine in response to ischemia [58]. Although CXCL6 was not identified by proteome analysis, an enrichment in proteins related to HIF-1α signaling, and CCR3 signaling, a pathway important for cell homing to inflammation and injury sites [50], was also found in hCPCs upon ischemia (Fig. 7a). Moreover, we were able to identify proteins associated with CXCR4 signaling, a pathway activated by SDF-1 with documented roles in cell motility and chemotactic response [59]. Altogether, our data and previous reports regarding CPC secretome, namely CXCL6 function, point to an effect of this chemokine in hCPC migration and angiogenesis. In fact, we also demonstrate pro-angiogenic properties of conditioned medium from the ischemic phase of injury.

IGF-1 is one of the key proteins shown to be upregulated upon injury or stress by CPCs [38, 60] and CMs [46, 49] and has also been shown to have a role in the activation of hCPC regenerative potential [37, 55] and proliferation [46, 49] in vitro and in vivo. Although no significant increase in IGF-1 secretion was detected by ELISA, we registered an enrichment in IGF-signaling associated proteins in hCPC proteome upon ischemia and reperfusion time points (Fig. 7a, c). Several other proteins associated with other cytokine/growth factor paracrine signaling pathways were also found to be more represented in hCPC proteome upon ischemia, including VEGF, IL-2, IL-3, IL-15, and GM-CSF signaling, all with documented paracrine roles acting in CM physiology during I/R injury [45, 61, 62].

hCPC proteome upon ischemia was also enriched in terms related with ROS metabolism, cysteine synthesis, glutamate metabolism, and glutathione-mediated detoxification (Fig. 7b). l-cysteine is one of the main precursors for glutathione, a molecule that protects cells from oxidative stress [51]. In fact, glutaminolysis, a pathway that leads to the production of glutathione via l-cysteine and glutamate, was found as a key energy source for proliferating mouse CPCs [63]. ROS formation and oxidative stress has been described as caused by the sudden oxygen elevation in the early reperfusion phase of injury [2], rather than during ischemia. However, during the preparation of the samples for proteomic analysis upon ischemia, hCPCs were briefly subjected to atmospheric oxygen concentrations, which could induce such oxidative stress responses. Together, these data point to a mechanistic response of hCPCs to ischemia phase of injury focused on a downregulation of cell cycle checkpoint and DNA repair processes in favor of an enrichment in proteins associated with stress coping mechanisms, induction of cell homing and motility, and activation of pathways associated with paracrine communication including CXCL6 secretion (Fig. 7a). Such paracrine signaling activation is described as having a beneficial effect on CM protection. hCPC paracrine protection on hiPSC-CM was recapitulated in our model. Viability of hiPSC-CMs was mainly affected in the early reperfusion phase of the injury, which is consistent with the described in vivo pathophysiology of CM death during I/R, where the first minutes of reperfusion are also the main trigger for CM death and tissue damage due to the oxidative stress, calcium overload, mitochondrial permeability pore opening, and hypercontracture [2].

Several authors have reported an increase in the number of resident CPC upon AMI in animal model [38, 54] hearts. Stastna et al., in 2010, also showed that connective tissue growth factor, a pro-fibrosis factor released by CMs upon AMI, induces rat CPC proliferation in vitro and atrial natriuretic peptide, a vasodilator molecule also secreted by CMs, had an opposite effect, decreasing rat CPC proliferation [64]. These results suggest that CPC proliferation is regulated upon injury by paracrine factors secreted by CMs. In fact, in our co-culture I/R injury model, we were able to observe a significant increase in hCPC proliferation upon reperfusion (Fig. 7b, c). Our hCPC whole proteome results also showed higher enrichment of pathways related with cell proliferation via EGF signaling after reperfusion, which points to EGF as one of the key actuator signals in CPC proliferation activation upon AMI (Fig. 7b, c). In fact, EGF has been reported as having a positive effect on cardiosphere-derived hCPCs proliferation and migration [65], as being one of the main factors secreted in mice CPC conditioned medium [55], as upregulated in mice CPCs upon injury [38], and EGF receptor has also been identified in a hCPC receptome characterization study by our group [19]. Other signaling pathways described to be related with cell proliferation were also found to be more enriched at 16 h post reperfusion, such as FLT3 signaling (Fig. 7c), a pathway described as important for proliferation in hematopoietic progenitor cells [66], cytokinesis, and IGF-1 pathway.

Collectively, our data suggests an hCPC response to early reperfusion through activation of paracrine signaling mechanisms (including acute phase response signaling), cell proliferation, glutathione-mediated ROS detoxification, and cell movement, while still inhibiting cell cycle repair mechanisms (Fig. 7b). Later in reperfusion, hCPC proteome also demonstrated an enrichment in several pathways associated with cytokine/growth factor paracrine signaling, all with documented paracrine roles acting in CM physiology during I/R injury, including CXCL6 secretion, a recovery of cell cycle repair mechanisms, and activation of angiogenesis and vasculogenesis-related pathways (Fig. 7c). These results are in line with the concept that hCPC regenerative capacity is mainly centered on their paracrine potential, being CXCL6 one of its critical players.

Our data support the importance of a paracrine crosstalk between CPCs and CMs during AMI, and the idea that heterotypic cell models better recapitulate in vivo features of I/R when compared to monotypic models.

## Conclusions

The I/R injury model developed recapitulates important hallmarks of AMI. The use of robust advanced analytical technologies such as LC-MS/MS whole cell proteome analysis, combined with more classical and targeted methodologies (ELISA growth factor quantification, viability assays) and functional assays (tube formation assay), enabled us to further understand the response of hCPC to I/R injury, shedding new insights on the possible mechanisms involved. We show, for the first time, an increase in CXCL6 secretion by hCPCs in a myocardial I/R setting, reinforcing the described role of CXCL6 in hCPC regenerative and pro-angiogenic potential and suggesting the importance of this chemokine in the paracrine-mediated protective effect of CPCs in the myocardium upon AMI. The results reported herein strengthen the importance of studying physiological processes using more complex human co-culture in vitro models leading to a better recapitulation of the in vivo paracrine signaling and a more relevant model for future applications such as drug discovery.

This work provides new insights and raises new questions related with hCPC biology in acute myocardial infarction. We believe that our model provides an important tool towards a better understanding of hCPC action mechanisms upon AMI, which will enable the development of novel therapies focused on activation, recruitment, and improvement of the endogenous heart regeneration capacity.

## Additional files

Additional file 1: **Figure S1.** Phenotypic characterization of hCPCs. hCPCs were characterized using specific cell markers by flow cytometry (A) and immunostaining (B). Scale bars: 50 μm. Error bars represent SD of *n* = 3 (unpaired *t* test). (PPTX 662 kb)

Additional file 2: **Figure S2.** Effect of I/R injury on hiPSCs-hCMs without maturation step. Viability of hiPSC-CMs after 15 days of differentiation (without maturation step) was assessed by PrestoBlue® assay (A) and by cell staining with FDA (live cells, green) and PI (dead cells, red), scale bars 200 μm (B). Black circles: mono-culture hiPSC-CMs CTL (M CM CTL); Red squares: mono-culture hiPSC-CMs insult (M CM i); Green triangles: co-culture hiPSC-CM insult (Co CM i). Post R: Post Reperfusion. (PPTX 1304 kb)

Additional file 3: **Figure S3.** Phenotypic characterization of hiPSC-CMs. hiPSC-CMs were characterized using specific cell markers by flow cytometry (A) and immunostaining (B). hiPSC-CMs retain their cardiomyocyte markers expression after 2 days in assay conditions (expansion medium at 3% O_2_: light gray bars) comparing to the hiPSC-CM maturation culture conditions (Pluricyte® CM medium at 21% O_2_: dark gray bars). Scale bars: 50 μm. Error bars represent SD of *n* = 3 (unpaired *t* test). (PPTX 791 kb)

Additional file 4: Top IPA Canonical Pathways, Diseases and Bio Functions identified. (XLSX 156 kb)

Additional file 5: **Figure S4.** Proteins identified in hCPCs. Venn diagram illustrates the overlap between proteins identified in hCPCs in: mono-culture control (M CPC CTL); co-culture control (Co CPC CTL); mono-culture insult (M CPC i), and co-culture insult (Co CPC i) conditions. Proteins related with cell proliferation, cytoskeleton organization, maintenance of cell integrity, cell death, paracrine signaling, regeneration, stress response, and metabolism are highlighted for the subset of proteins identified exclusively in Co CPC i proteome. (PPTX 312 kb)

Additional file 6: **Table S1.** Canonical pathways and functions enriched in Co CPC I vs Co CPC CTL. –log (*p* value) ≤ 1.3 were considered as non-significant (n.s.) (less than 95% confidence). Pathway/ function terms were only selected for analysis when –log (*p* value) ratio between the two conditions ≥ 1.2. (DOCX 26 kb)

Additional file 7: **Table S2.** Canonical pathways and functions enriched in co CPC I vs mono CPC i. –log (*p* value) ≤ 1.3 were considered as non-significant (n.s.) (less than 95% confidence). Pathway/ function terms were only selected for analysis when –log (*p* value) ratio between the two conditions ≥ 1.2 (DOCX 27 kb)

Additional file 8: Canonical pathways and functions differentially enriched in Co CPC CTL and Co CPC throughout injury. (DOCX 37 kb)

## Abbreviations

AMI: Acute myocardial infarction
bFGF: Basic fibroblast growth factor
BI: Before injury
CHF: Chronic heart failure
CMs: Cardiomyocytes
Co CTL: Co-culture control
Co i: Co-culture injury
CPCs: Cardiac progenitor cells
CXCL6: Granulocyte chemotactic protein 2
DPBS: Dulbecco’s phosphate-buffered saline
EGF: Epidermal growth factor
ELISA: Enzyme-linked immunosorbent assay
ExpM: Expansion medium
FBS: Fetal bovine serum
FDA: Fluorescein diacetate
FSG: Fish skin gelatin
hCPCs: Human cardiac progenitor cells
HGF: Hepatocyte growth factor
hiPSC-CMs: Human-induced pluripotent stem cell-derived cardiomyocyte
HUVECs: Human umbilical vein endothelial cells
I/R: Ischemia/reperfusion
IGF-1: Insulin-like growth factor 1
IMS: Ischemic mimetic solution
IPA: Ingenuity pathway analysis
LC: Liquid chromatography
M CTL: Mono-culture control
M i: Mono-culture injury
MS: Mass spectrometry
PFA: Paraformaldehyde
PI: Propidium iodide
Post I: Post ischemia
Post R: Post reperfusion
