# Supplementary figures and images for: Human cardiac progenitor cell activation and regeneration mechanisms: exploring a novel myocardial ischemia/reperfusion in vitro model
Source: Stem Cell Res Ther. 2019 Mar 7;10:77. doi: 10.1186/s13287-019-1174-4 (PMC6407246; doi:10.1186/s13287-019-1174-4)

## Slide 1
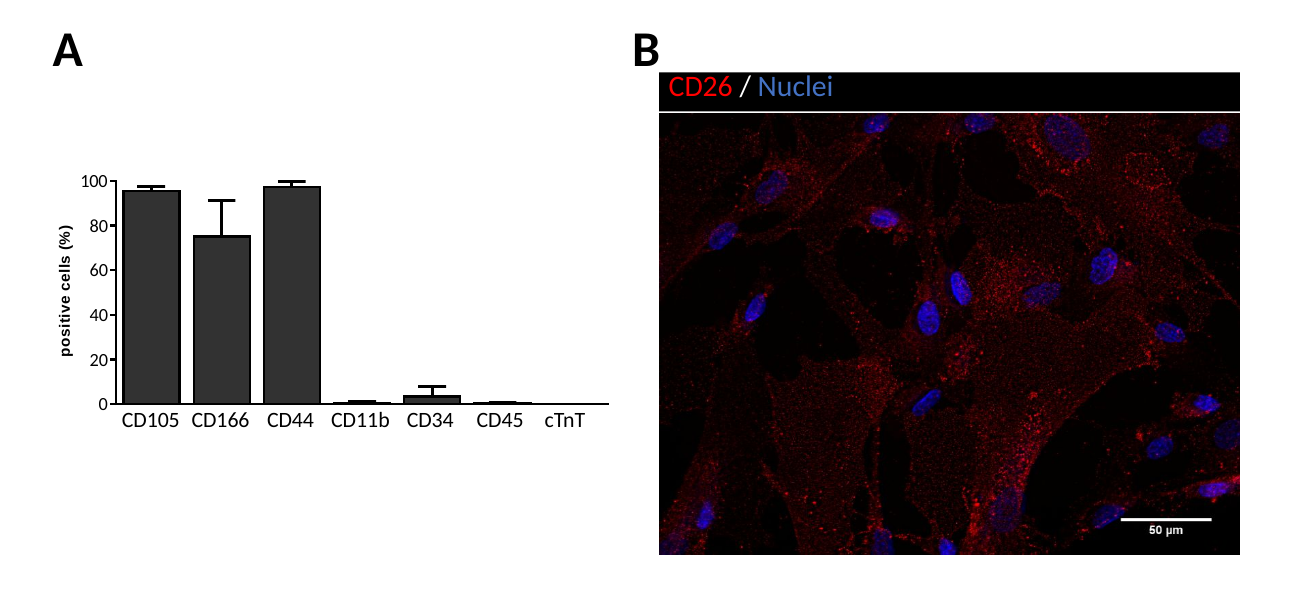

A
B
CD26 / Nuclei
CD105
CD166
CD44
CD11b
CD34
CD45
cTnT

Supplement: Supplementary file 1 — Figure S1. Phenotypic characterization of hCPCs. hCPCs were characterized using specific cell markers by flow cytometry (A) and immunostaining (B). Scale bars: 50 μm. Error bars represent SD of n = 3 (unpaired t test). (PPTX 662 kb) [file 13287_2019_1174_MOESM1_ESM.pptx]

## Slide 1
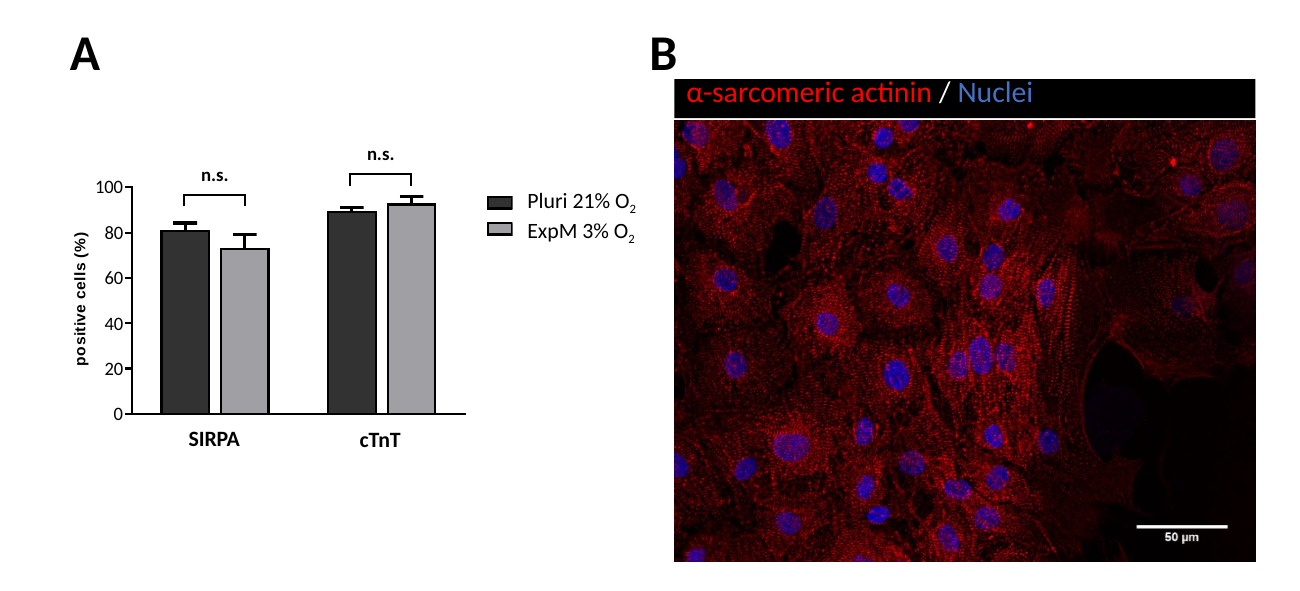

A
B
α-sarcomeric actinin / Nuclei
n.s.
n.s.
Pluri 21% O2
ExpM 3% O2
SIRPA
cTnT

Supplement: Supplementary file 3 — Figure S3. Phenotypic characterization of hiPSC-CMs. hiPSC-CMs were characterized using specific cell markers by flow cytometry (A) and immunostaining (B). hiPSC-CMs retain their cardiomyocyte markers expression after 2 days in assay conditions (expansion medium at 3% O2: light gray bars) comparing to the hiPSC-CM maturation culture conditions (Pluricyte® CM medium at 21% O2: dark gray bars). Scale bars: 50 μm. Error bars represent SD of n = 3 (unpaired t test). (PPTX 791 kb) [file 13287_2019_1174_MOESM3_ESM.pptx]
