# Supplementary material for: Human cardiac progenitor cell activation and regeneration mechanisms: exploring a novel myocardial ischemia/reperfusion in vitro model
Source: Stem Cell Res Ther. 2019 Mar 7;10:77. doi: 10.1186/s13287-019-1174-4 (PMC6407246; doi:10.1186/s13287-019-1174-4)
Supplement: Supplementary file 2 — Figure S2. Effect of I/R injury on hiPSCs-hCMs without maturation step. Viability of hiPSC-CMs after 15 days of differentiation (without maturation step) was assessed by PrestoBlue® assay (A) and by cell staining with FDA (live cells, green) and PI (dead cells, red), scale bars 200 μm (B). Black circles: mono-culture hiPSC-CMs CTL (M CM CTL); Red squares: mono-culture hiPSC-CMs insult (M CM i); Green triangles: co-culture hiPSC-CM insult (Co CM i). Post R: Post Reperfusion. (PPTX 1304 kb) [file 13287_2019_1174_MOESM2_ESM.pptx]

## Slide 1
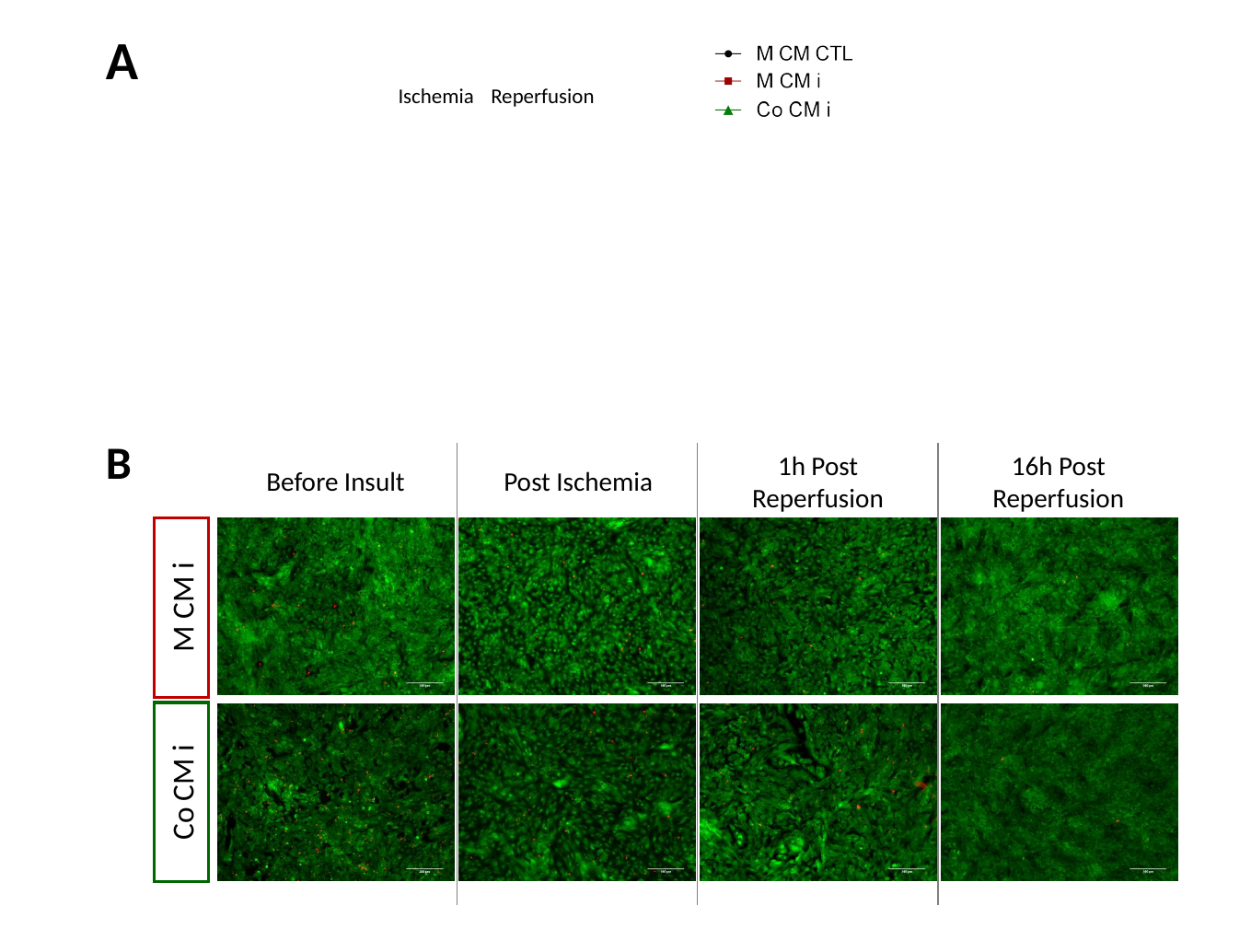

A
Ischemia
Reperfusion
B
1h Post Reperfusion
16h Post Reperfusion
Before Insult
Post Ischemia
M CM i
Co CM i
