# Supplementary material for: Human cardiac progenitor cell activation and regeneration mechanisms: exploring a novel myocardial ischemia/reperfusion in vitro model
Source: Stem Cell Res Ther. 2019 Mar 7;10:77. doi: 10.1186/s13287-019-1174-4 (PMC6407246; doi:10.1186/s13287-019-1174-4)
Supplement: Supplementary file 5 — Figure S4. Proteins identified in hCPCs. Venn diagram illustrates the overlap between proteins identified in hCPCs in: mono-culture control (M CPC CTL); co-culture control (Co CPC CTL); mono-culture insult (M CPC i), and co-culture insult (Co CPC i) conditions. Proteins related with cell proliferation, cytoskeleton organization, maintenance of cell integrity, cell death, paracrine signaling, regeneration, stress response, and metabolism are highlighted for the subset of proteins identified exclusively in Co CPC i proteome. (PPTX 312 kb) [file 13287_2019_1174_MOESM5_ESM.pptx]

## Slide 1
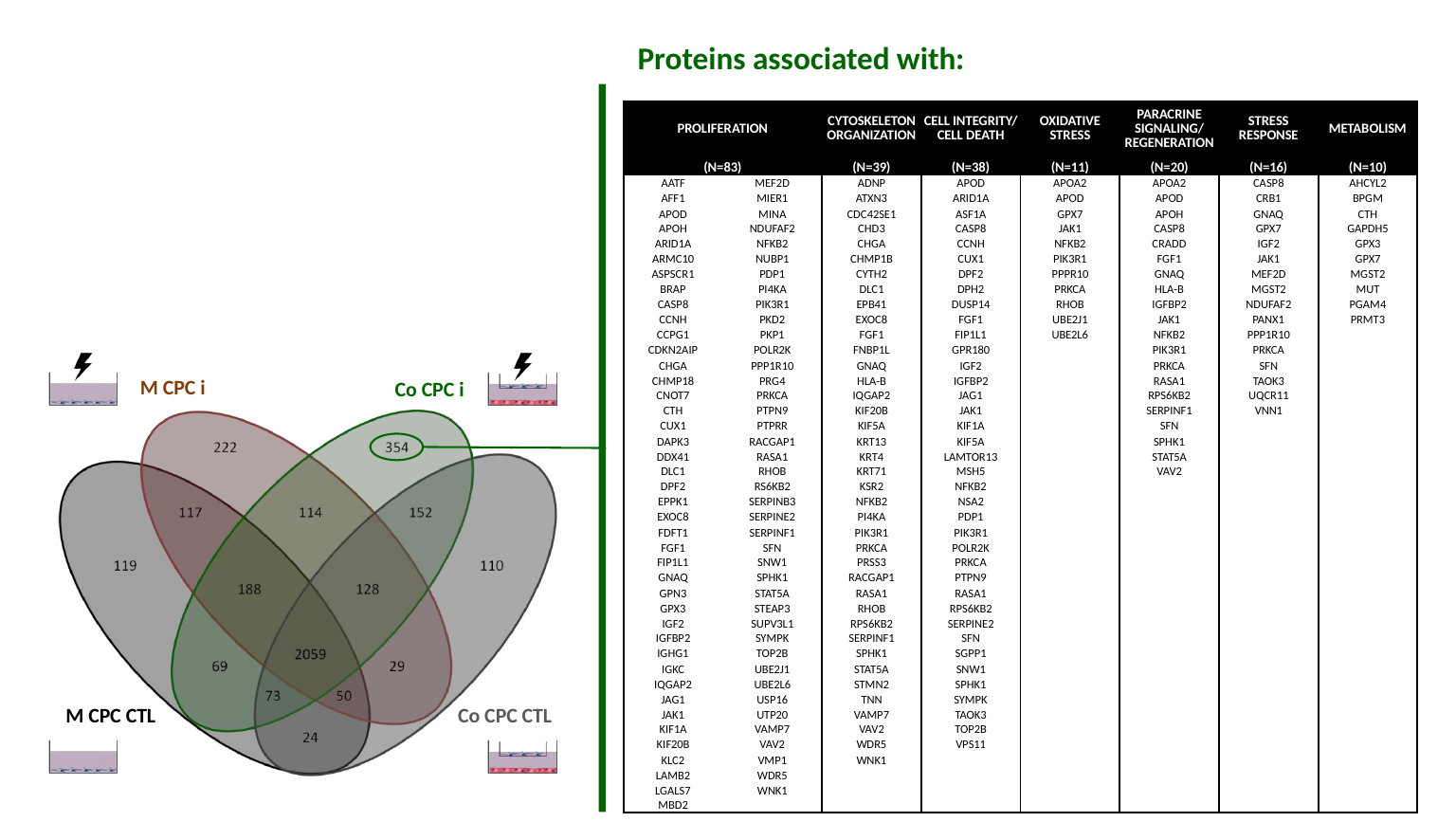

Proteins associated with:
| PROLIFERATION | | CYTOSKELETON ORGANIZATION | CELL INTEGRITY/ CELL DEATH | OXIDATIVE STRESS | PARACRINE SIGNALING/ REGENERATION | STRESS RESPONSE | METABOLISM |
| --- | --- | --- | --- | --- | --- | --- | --- |
| (N=83) | | (N=39) | (N=38) | (N=11) | (N=20) | (N=16) | (N=10) |
| AATF | MEF2D | ADNP | APOD | APOA2 | APOA2 | CASP8 | AHCYL2 |
| AFF1 | MIER1 | ATXN3 | ARID1A | APOD | APOD | CRB1 | BPGM |
| APOD | MINA | CDC42SE1 | ASF1A | GPX7 | APOH | GNAQ | CTH |
| APOH | NDUFAF2 | CHD3 | CASP8 | JAK1 | CASP8 | GPX7 | GAPDH5 |
| ARID1A | NFKB2 | CHGA | CCNH | NFKB2 | CRADD | IGF2 | GPX3 |
| ARMC10 | NUBP1 | CHMP1B | CUX1 | PIK3R1 | FGF1 | JAK1 | GPX7 |
| ASPSCR1 | PDP1 | CYTH2 | DPF2 | PPPR10 | GNAQ | MEF2D | MGST2 |
| BRAP | PI4KA | DLC1 | DPH2 | PRKCA | HLA-B | MGST2 | MUT |
| CASP8 | PIK3R1 | EPB41 | DUSP14 | RHOB | IGFBP2 | NDUFAF2 | PGAM4 |
| CCNH | PKD2 | EXOC8 | FGF1 | UBE2J1 | JAK1 | PANX1 | PRMT3 |
| CCPG1 | PKP1 | FGF1 | FIP1L1 | UBE2L6 | NFKB2 | PPP1R10 | |
| CDKN2AIP | POLR2K | FNBP1L | GPR180 | | PIK3R1 | PRKCA | |
| CHGA | PPP1R10 | GNAQ | IGF2 | | PRKCA | SFN | |
| CHMP18 | PRG4 | HLA-B | IGFBP2 | | RASA1 | TAOK3 | |
| CNOT7 | PRKCA | IQGAP2 | JAG1 | | RPS6KB2 | UQCR11 | |
| CTH | PTPN9 | KIF20B | JAK1 | | SERPINF1 | VNN1 | |
| CUX1 | PTPRR | KIF5A | KIF1A | | SFN | | |
| DAPK3 | RACGAP1 | KRT13 | KIF5A | | SPHK1 | | |
| DDX41 | RASA1 | KRT4 | LAMTOR13 | | STAT5A | | |
| DLC1 | RHOB | KRT71 | MSH5 | | VAV2 | | |
| DPF2 | RS6KB2 | KSR2 | NFKB2 | | | | |
| EPPK1 | SERPINB3 | NFKB2 | NSA2 | | | | |
| EXOC8 | SERPINE2 | PI4KA | PDP1 | | | | |
| FDFT1 | SERPINF1 | PIK3R1 | PIK3R1 | | | | |
| FGF1 | SFN | PRKCA | POLR2K | | | | |
| FIP1L1 | SNW1 | PRSS3 | PRKCA | | | | |
| GNAQ | SPHK1 | RACGAP1 | PTPN9 | | | | |
| GPN3 | STAT5A | RASA1 | RASA1 | | | | |
| GPX3 | STEAP3 | RHOB | RPS6KB2 | | | | |
| IGF2 | SUPV3L1 | RPS6KB2 | SERPINE2 | | | | |
| IGFBP2 | SYMPK | SERPINF1 | SFN | | | | |
| IGHG1 | TOP2B | SPHK1 | SGPP1 | | | | |
| IGKC | UBE2J1 | STAT5A | SNW1 | | | | |
| IQGAP2 | UBE2L6 | STMN2 | SPHK1 | | | | |
| JAG1 | USP16 | TNN | SYMPK | | | | |
| JAK1 | UTP20 | VAMP7 | TAOK3 | | | | |
| KIF1A | VAMP7 | VAV2 | TOP2B | | | | |
| KIF20B | VAV2 | WDR5 | VPS11 | | | | |
| KLC2 | VMP1 | WNK1 | | | | | |
| LAMB2 | WDR5 | | | | | | |
| LGALS7 | WNK1 | | | | | | |
| MBD2 | | | | | | | |
M CPC i
Co CPC i
M CPC CTL
Co CPC CTL
