# Supplementary material for: Human cardiac progenitor cell activation and regeneration mechanisms: exploring a novel myocardial ischemia/reperfusion in vitro model
Source: Stem Cell Res Ther. 2019 Mar 7;10:77. doi: 10.1186/s13287-019-1174-4 (PMC6407246; doi:10.1186/s13287-019-1174-4)
Supplement: Supplementary file 6 — Table S1. Canonical pathways and functions enriched in Co CPC I vs Co CPC CTL. –log (p value) ≤ 1.3 were considered as non-significant (n.s.) (less than 95% confidence). Pathway/ function terms were only selected for analysis when –log (p value) ratio between the two conditions ≥ 1.2. (DOCX 26 kb) [file 13287_2019_1174_MOESM6_ESM.docx]

**Additional file 6: Table S1. Canonical pathways and functions enriched in Co CPC I vs Co CPC CTL.**  –log (p-value)≤1.3 were considered as non significant (n.s.) (less than 95% confidence). Pathway/ function terms were only selected for analysis when –log (p-value) ratio between the two conditions ≥1.2.

|  |  | **-log (p-value)** | |
| --- | --- | --- | --- |
| **Category** | **Canonical Pathway/ Function** | **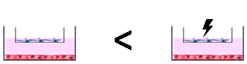Co CPC CTL** | **Co CPC i** |
|  |  |  |  |
| **Cell Proliferation** | Cholecystokinin/Gastrin-mediated Signaling | 1.60 | 3.09 |
|  | EGF Signaling | n.s. | 2.82 |
|  | Renin-Angiotensin Signaling | 1.61 | 2.52 |
|  | ERK5 Signaling | n.s. | 2.48 |
|  | FLT3 Signaling in Hematopoietic Progenitor Cells | n.s. | 1.80 |
|  |  |  |  |
| **Cytoskeleton Organization** | Quantity of actin stress fibers | n.s. | 5.32 |
|  | Quantity of actin filaments | n.s. | 5.19 |
|  | Quantity of filaments | n.s. | 5.08 |
|  |  |  |  |
| **Maintenance of Cell Integrity / Cell Death** | DNA Methylation and Transcriptional Repression Signaling | 2.55 | 4.17 |
|  | Death Receptor Signaling | 2.79 | 3.54 |
|  | Myc Mediated Apoptosis Signaling | 2.39 | 3.04 |
|  | Autophagy | n.s. | 1.53 |
|  |  |  |  |
| **Oxidative Stress** | Hypoxia Signaling in the Cardiovascular System | 2.94 | 4.25 |
|  | Superoxide Radicals Degradation | 1.85 | 2.43 |
|  |  |  |  |
| **Paracrine Signaling / Regeneration** | VEGF Signaling | 5.92 | 7.14 |
|  | Differentiation of Cells | n.s. | 5.60 |
|  | Oncostatin M Signaling | 4.25 | 5.41 |
|  | Vasculogenesis | n.s. | 5.10 |
|  | PDGF Signaling | 2.50 | 4.04 |
|  | IL-3 Signaling | 2.25 | 3.07 |
|  | IL-2 Signaling | n.s. | 2.03 |
|  | JAK/Stat Signaling | 1.34 | 2.70 |
|  | PEDF Signaling | n.s. | 1.42 |
|  | Role of JAK family kinases in IL-6-type Cytokine Signaling | n.s. | 1.46 |
|  | IL-1 Signaling | 1.52 | 1.85 |
|  |  |  |  |
| **Stress Response** | Endoplasmic reticulum stress response | 10.35 | 12.49 |
|  | Tec Kinase Signaling | 2.59 | 4.31 |
|  | PDGF Signaling | 2.50 | 4.04 |
|  | AMPK Signaling | 3.03 | 3.93 |
|  | Acute Myeloid Leukemia Signaling | n.s. | 1.91 |
|  | Corticotropin Releasing Hormone Signaling | n.s. | 1.32 |
|  |  |  |  |
| **Metabolism** | Glutathione Redox Reactions I | 3.06 | 5.63 |
|  | Glutathione-mediated Detoxification | 1.99 | 3.27 |
|  | Cysteine Biosynthesis/Homocysteine Degradation | n.s. | 1.60 |
|  | Cysteine Biosynthesis III (mammalia) | 2.20 | 3.89 |
|  | Methionine Degradation I (to Homocysteine) | 1.86 | 2.80 |
