# Supplementary material for: Human cardiac progenitor cell activation and regeneration mechanisms: exploring a novel myocardial ischemia/reperfusion in vitro model
Source: Stem Cell Res Ther. 2019 Mar 7;10:77. doi: 10.1186/s13287-019-1174-4 (PMC6407246; doi:10.1186/s13287-019-1174-4)
Supplement: Supplementary file 7 — Table S2. Canonical pathways and functions enriched in co CPC I vs mono CPC i. –log (p value) ≤ 1.3 were considered as non-significant (n.s.) (less than 95% confidence). Pathway/ function terms were only selected for analysis when –log (p value) ratio between the two conditions ≥ 1.2 (DOCX 27 kb) [file 13287_2019_1174_MOESM7_ESM.docx]

**Additional file 7: Table S2. Canonical pathways and functions enriched in Co CPC I vs Mono CPC i.**  –log (p-value)≤1.3 were considered as non significant (n.s.) (less than 95% confidence). Pathway/ function terms were only selected for analysis when –log (p-value) ratio between the two conditions ≥1.2.

|  |  | **-log (p-value)** | |
| --- | --- | --- | --- |
| **Category** | **Canonical Pathway/ Function** | **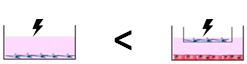Mono CPC i** | **Co CPC i** |
|  |  |  |  |
| **Cell Proliferation** | Mitosis | 5.34 | 7.49 |
|  | Cholecystokinin/Gastrin-mediated Signaling | 1.99 | 3.09 |
|  | EGF Signaling | 1.46 | 2.82 |
|  | Renin-Angiotensin Signaling | 1.90 | 2.52 |
|  | ERK5 Signaling | n.s. | 2.48 |
|  | Prolactin Signaling | n.s. | 1.47 |
|  |  |  |  |
| **Cytoskeleton Organization** | Morphology of Cells | 18.76 | 23.54 |
|  | Cdc42 Signaling | 6.64 | 9.00 |
|  | Quantity of Actin Stress Fibers | 4.42 | 5.32 |
|  | Quantity of Actin Filaments | 3.97 | 5.19 |
|  | PTEN Signaling | 2.76 | 3.48 |
|  |  |  |  |
| **Maintenance of Cel\l Integrity/ Cell Death** | Myc Mediated Apoptosis Signaling | 2.32 | 3.04 |
|  | BER pathway | n.s. | 2.20 |
|  |  |  |  |
| **Oxidative Stress** | Metabolism of ROS | 5.45 | 7.63 |
|  | Synthesis of ROS | 4.83 | 6.65 |
|  | Superoxide Radicals Degradation | 1.70 | 2.43 |
|  |  |  |  |
| **Paracrine Signaling / Regeneration** | IGF-1 Signaling | 4.06 | 6.87 |
|  | Oncostatin M Signaling | 4.45 | 5.41 |
|  | vasculogenesis | n.s. | 5.10 |
|  | Chemokine Signaling | 3.74 | 4.63 |
|  | Neuregulin Signaling | 2.26 | 4.44 |
|  | p70S6K Signaling | 3.36 | 4.04 |
|  | PDGF Signaling | 2.67 | 4.04 |
|  | IL-3 Signaling | 1.81 | 3.07 |
|  | HGF Signaling | 1.88 | 2.84 |
|  | JAK/Stat Signaling | 1.52 | 2.70 |
|  | IL-2 Signaling | 1.38 | 2.03 |
|  | IL-1 Signaling | 1.41 | 1.85 |
|  | Netrin Signaling | n.s. | 1.82 |
|  | CNTF Signaling | 1.31 | 1.64 |
|  | TNFR1 Signaling | n.s. | 1.55 |
|  | Role of JAK family kinases in IL-6-type Cytokine Signaling | n.s. | 1.46 |
|  | PEDF Signaling | n.s. | 1.42 |
|  |  |  |  |
| **Stress Response** | Corticotropin Releasing Hormone Signaling | n.s. | 1.32 |
|  | Acute Phase Response Signaling | 2.95 | 3.99 |
|  |  |  |  |
| **Metabolism** | Cysteine Biosynthesis/Homocysteine Degradation | n.s. | 1.60 |
|  | Cysteine Biosynthesis III (mammalia) | 1.98 | 3.89 |
|  | Methionine Degradation I (to Homocysteine) | 1.67 | 2.80 |
|  | Glycolysis I | 5.14 | 7.48 |
|  | Glycolysis of cells | n.s. | 5.17 |
