# Supplementary material for: Human cardiac progenitor cell activation and regeneration mechanisms: exploring a novel myocardial ischemia/reperfusion in vitro model
Source: Stem Cell Res Ther. 2019 Mar 7;10:77. doi: 10.1186/s13287-019-1174-4 (PMC6407246; doi:10.1186/s13287-019-1174-4)
Supplement: Supplementary file 8 — Canonical pathways and functions differentially enriched in Co CPC CTL and Co CPC throughout injury. (DOCX 37 kb) [file 13287_2019_1174_MOESM8_ESM.docx]

**Table 1. Canonical pathways and functions differentially enriched in Co CPC CTL and Co CPC throughout injury.**

|  |  | **-log (p-value)** **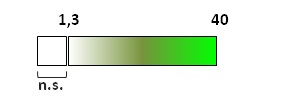** | | | |
| --- | --- | --- | --- | --- | --- |
| **Category** | **Canonical Pathway/ Function** | **Co CPC CTL** | **Co CPC Post I** | **Co CPC 1h Post R** | **Co CPC 16h Post R** |
|  |  |  |  |  |  |
|  |  |  |  |  |  |
|  |  |  |  |  |  |
| **1. Cell Proliferation** | Cell Cycle Progression | 11.82 | 8.58 | 7.41 | 9.96 |
|  | Cytokinesis | n.s. | 4.35 | n.s. | n.s. |
|  | Mitosis | 7.39 | 4.97 | n.s. | 6.88 |
|  | Arrest in Mitosis | 4.78 | n.s. | n.s. | 5.38 |
|  | Role of CHK Proteins in Cell Cycle Checkpoint Control | 2.33 | n.s. | n.s. | 1.74 |
|  | Cell Cycle: G1/S Checkpoint Regulation | 1.44 | n.s. | n.s. | 1.91 |
|  | Cell Cycle Regulation by BTG Family Proteins | 1.43 | n.s. | n.s. | 2.16 |
|  | ERK5 Signaling | n.s. | 1.49 | 2.13 | 2.67 |
|  | EGF Signaling | n.s. | n.s. | 1.48 | 2.67 |
|  | Cholecystokinin/Gastrin-mediated Signaling | 1.60 | 2.01 | 2.50 | 3.21 |
|  | FLT3 Signaling in Hematopoietic Progenitor Cells | n.s. | n.s. | n.s. | 1.46 |
|  |  |  |  |  |  |
| **2. Cytoskeleton Organization** | Cell Movement of Embryonic Cell Lines | n.s. | n.s. | 5.83 | n.s. |
|  | Cell Movement of Epithelial Cell Lines | n.s. | n.s. | 4.85 | n.s. |
|  | Cell Movement of Kidney Cell Lines | n.s. | n.s. | 5.02 | n.s. |
|  | Extension of Cells | n.s. | 3.89 | n.s. | n.s. |
|  | Invasion of Tumor | n.s. | 4.59 | n.s. | n.s. |
|  | Invasion of Tumor Cells | n.s. | 3.98 | n.s. | n.s. |
|  | CCR3 Signaling in Eonisophils | 2.67 | 3.53 | 2.09 | 2.33 |
|  |  |  |  |  |  |
| **3. Maintenance of Cell Integrity / Cell Death** | Apoptosis | 35.06 | 26.94 | 29.56 | 36.25 |
|  | Necrosis | 36.61 | 28.54 | 33.19 | 36.68 |
|  | Autophagy | 5.85 | 4.04 | 5.01 | 5.75 |
|  | Cell Survival | 18.01 | 12.04 | 15.00 | 18.82 |
|  | Cell Viability | 17.77 | 11.82 | 15.53 | 18.72 |
|  | Death Receptor Signaling | 2.79 | 1.52 | 3.56 | 2.48 |
|  | Repair of DNA | 5.70 | n.s. | 5.69 | 7.07 |
|  | Nucleotide Excision Repair Pathway | 1.43 | n.s. | n.s. | n.s. |
|  | Cell Cycle: G2/M DNA Damage Checkpoint Regulation | 1.60 | n.s. | 1.60 | 2.20 |
|  | Mismatch Repair in Eukaryotes | 3.32 | n.s. | 2.54 | 3.16 |
|  | BER Pathway | 2.59 | n.s. | 1.83 | 2.47 |
|  |  |  |  |  |  |
| **4. Oxidative Stress** | Hypoxia Signaling in the Cardiovascular System | 2.94 | 3.14 | 3.40 | 3.56 |
|  | Synthesis of ROS | 7.82 | 7.27 | 6.09 | 7.19 |
|  | Metabolism of ROS | 9.06 | 7.97 | 7.24 | 8.34 |
|  | Superoxide Radicals Degradation | 1.85 | 2.20 | 2.78 | 2.67 |
|  | Metabolism of H_2_O_2_ | n.s. | 4.42 | n.s. | n.s. |
|  | Catabolism of H_2_O_2_ | n.s. | 4.12 | n.s. | n.s. |
|  | HIF1a Signaling | n.s. | 1.53 | n.s. | n.s. |
|  |  |  |  |  |  |
| **5. Paracrine Signaling / Regeneration** | IGF-1 Signaling | 4.00 | 6.43 | 5.78 | 5.73 |
|  | GM-CSF Signaling | 1.84 | 2.43 | 1.53 | 1.63 |
|  | HGF Signaling | 3.06 | 2.72 | 2.10 | 3.03 |
|  | VEGF Signaling | 5.92 | 7.04 | 5.92 | 7.46 |
|  | IL-2 Signaling | n.s. | 1.79 | n.s. | 1.84 |
|  | IL-3 Signaling | 2.25 | 2.61 | 1.92 | 3.06 |
|  | IL-8 Signaling | 5.45 | 4.76 | 3.78 | 3.55 |
|  | IL-15 Signaling | n.s. | 1.54 | 1.38 | 1.77 |
|  | IL-22 Signaling | n.s. | n.s. | n.s. | 1.37 |
|  | Role of JAK family kinases in IL-6-type Cytokine Signaling | n.s. | n.s. | n.s. | 1.75 |
|  | JAK/Stat Signaling | 1.34 | n.s. | 1.61 | 1.99 |
|  | PDGF Signaling | 2.50 | 2.58 | 3.24 | 4.12 |
|  | Neuregulin Signaling | 3.99 | 3.24 | 2.40 | 4.50 |
|  | Oncostatin M Signaling | 4.25 | 3.92 | 3.00 | 5.39 |
|  | Vasculogenesis | n.s. | n.s. | n.s. | 4.94 |
|  | Angiogenesis | 7.06 | 5.55 | 6.28 | 7.35 |
|  | Differentiation of Cells | n.s. | 4.25 | 4.73 | 6.45 |
|  |  |  |  |  |  |
| **6. Stress Response** | Hypersensitive Reaction | 7.83 | 10.84 | 7.50 | 8.03 |
|  | Acute Phase Response Signaling | 3.47 | 3.77 | 4.11 | 2.73 |
|  | Stress Response of Cells | n.s. | n.s. | 4.64 | n.s. |
|  | Endoplasmic Reticulum Stress Pathway | n.s. | 1.72 | n.s. | 1.68 |
|  | Unfolded Protein Response | 2.50 | 3.00 | 1.70 | 1.88 |
|  | Cardiac ß-adrenergic Signaling | 1.66 | 2.65 | n.s. | n.s. |
|  | Corticotropin Releasing Hormone Signaling | n.s. | n.s. | n.s. | 1.62 |
|  | HIPPO signaling | 5.54 | 4.23 | 5.03 | 5.08 |
|  |  |  |  |  |  |
| **7. Metabolism** | Glutathione Redox Reactions I | 3.06 | 3.03 | 6.52 | 3.61 |
|  | Glutathione-mediated Detoxification | 1.99 | 2.60 | 3.95 | 2.42 |
|  | Cysteine Biosynthesis III (mammalia) | 2.20 | 3.57 | 2.20 | 2.74 |
|  | Methionine Degradation I (to Homocysteine) | 1.86 | 2.35 | 1.86 | 2.41 |
|  | Cysteine Biosynthesis/Homocysteine Degradation | n.s. | 1.95 | n.s. | n.s. |
|  | Glutamate Biosynthesis II | n.s. | n.s. | 1.75 | 1.70 |
|  | Glutamate Degradation X | n.s. | n.s. | 1.75 | 1.70 |
|  | Glycolysis I | 5.60 | 7.79 | 5.60 | 7.21 |
|  | Glycolysis of cells | n.s. | 4.07 | n.s. | n.s. |
|  | Consumption of oxygen | n.s. | 4.30 | n.s. | n.s. |

log (p-value)≤1.3 were considered as non significant (n.s.) (less than 95% confidence).
